# Effects of Aberrant *Pax6* Gene Dosage on Mouse Corneal Pathophysiology and Corneal Epithelial Homeostasis

**DOI:** 10.1371/journal.pone.0028895

**Published:** 2011-12-29

**Authors:** Richard L. Mort, Adam J. Bentley, Francis L. Martin, J. Martin Collinson, Panagiotis Douvaras, Robert E. Hill, Steven D. Morley, Nigel J. Fullwood, John D. West

**Affiliations:** 1 Division of Reproductive and Developmental Sciences, University of Edinburgh, Edinburgh, United Kingdom; 2 Medical Research Council Human Genetics Unit, Medical Research Council Institute of Genetics and Molecular Medicine, University of Edinburgh, Edinburgh, United Kingdom; 3 Division of Biomedical and Life Sciences, Lancaster University, Lancaster, United Kingdom; 4 Centre for Biophotonics, Lancaster University, Lancaster, United Kingdom; 5 Institute of Medical Sciences, University of Aberdeen, Aberdeen, United Kingdom; 6 School of Clinical Sciences & Community Health, University of Edinburgh, Edinburgh, United Kingdom; University of Reading, United Kingdom

## Abstract

**Background:**

Altered dosage of the transcription factor PAX6 causes multiple human eye pathophysiologies. *PAX6* ^+/−^ heterozygotes suffer from aniridia and aniridia-related keratopathy (ARK), a corneal deterioration that probably involves a limbal epithelial stem cell (LESC) deficiency. Heterozygous *Pax6^+/Sey-Neu^* (*Pax6^+/−^*) mice recapitulate the human disease and are a good model of ARK. Corneal pathologies also occur in other mouse *Pax6* mutants and in *PAX77^Tg/−^* transgenics, which over-express Pax6 and model human *PAX6* duplication.

**Methodology/Principal Findings:**

We used electron microscopy to investigate ocular defects in *Pax6^+/−^* heterozygotes (low Pax6 levels) and *PAX77^Tg/−^* transgenics (high Pax6 levels). As well as the well-documented epithelial defects, aberrant *Pax6* dosage had profound effects on the corneal stroma and endothelium in both genotypes, including cellular vacuolation, similar to that reported for human macular corneal dystrophy. We used mosaic expression of an X-linked *LacZ* transgene in X-inactivation mosaic female (*XLacZ^Tg/−^*) mice to investigate corneal epithelial maintenance by LESC clones in *Pax6^+/−^* and *PAX77^Tg/−^* mosaic mice. *PAX77^Tg/−^* mosaics, over-expressing Pax6, produced normal corneal epithelial radial striped patterns (despite other corneal defects), suggesting that centripetal cell movement was unaffected. Moderately disrupted patterns in *Pax6^+/−^* mosaics were corrected by introducing the *PAX77* transgene (in *Pax6^+/−^*, *PAX77^Tg/−^* mosaics). *Pax6^Leca4/+^*, *XLacZ^Tg/−^* mosaic mice (heterozygous for the *Pax6^Leca4^* missense mutation) showed more severely disrupted mosaic patterns. Corrected corneal epithelial stripe numbers (an indirect estimate of active LESC clone numbers) declined with age (between 15 and 30 weeks) in wild-type *XLacZ^Tg/−^* mosaics. In contrast, corrected stripe numbers were already low at 15 weeks in *Pax6^+/−^* and *PAX77^Tg/−^* mosaic corneas, suggesting Pax6 under- and over-expression both affect LESC clones.

**Conclusions/Significance:**

*Pax6^+/−^* and *PAX77^Tg/−^* genotypes have only relatively minor effects on LESC clone numbers but cause more severe corneal endothelial and stromal defects. This should prompt further investigations of the pathophysiology underlying human aniridia and ARK.

## Introduction

The *Pax6* gene encodes the Pax6 transcription factor with key regulatory roles in eye development [1]–[5]. Abnormal expression results in a spectrum of ocular pathophysiologies, some of which are directly linked to the protein level [6]–[9]. Some corneal abnormalities associated with Pax6 mutations occur during development and others result from inadequate tissue maintenance.

It is widely accepted that, during adult corneal epithelial homeostasis, cell production is initiated by limbal epithelial stem cells (LESCs) in the limbus at the corneoscleral junction [10]–[14]. LESCs proliferate to produce transient (or transit) amplifying cells (TACs) that migrate centripetally, dividing a few times before terminally differentiating. As TACs differentiate they lose contact with the basal epithelium, move apically, and are desquamated from the surface layer [15], [16]. Epithelial abnormalities could be caused by defects in LESCs or epithelial cell proliferation, movement or loss.

Centripetal movement in the mouse corneal epithelium has been demonstrated directly in several experimental systems [17], [18] and indirectly by the postnatal switch from a randomly orientated mosaic pattern to radial stripes in X-inactivation mosaics [19]–[21] and lentivirus-labelled lineages [22]. Radial stripes emerging from the periphery and extending towards the central cornea from ∼5 weeks are thought to represent clones of centripetally migrating epithelial cells produced after LESC activation. Numerical analysis of these striping patterns provides an indirect estimate of the number of coherent clones of LESCs maintaining the corneal epithelium [19]–[21].

Pax6 is widely expressed during eye development [23] and continues in several adult tissues, including the corneal, limbal and conjunctival epithelia [24]. Absence of Pax6 causes anophthalmia in both mice [1] and humans [25]. Eye development is highly sensitive to Pax6 dose and haploinsufficiency in human *PAX6^+/−^* heterozygotes is characterised by aniridia and other ocular abnormalities [26]–[28]. Heterozygosity for mouse *Pax6^−^* null mutations, such as *Pax6^Sey^* [1], [29], [30], *Pax6^Sey-Neu^* [1], [31] and *Pax6^LacZ^* [32], causes similar abnormalities and small-eyes. Ocular phenotypes produced by hypomorphic *Pax6* alleles showed that surface ectoderm derivatives are more sensitive to Pax6 levels than optic vesicle derivatives [6]. *PAX6* missense mutations often cause different eye phenotypes from null mutations [33]–[35]. For example, *Pax6^Leca4/+^* heterozygous mice have small, abnormal eyes with corneal vascularisation from fetal stages and pigmentation (but no goblet cells) within the cornea [36].

*Pax6^+/−^* mice have corneal and other anterior segment abnormalities [37]–[46] and some have been characterised by electron microscopy (EM) [38], [47], [48]. The postnatal corneal deterioration in *Pax6^+/−^* mice is equivalent to that seen in human aniridia-related keratopathy (ARK), which has been attributed to LESC deficiency. This is based entirely on indirect evidence such as the presence of goblet cells [49] and clinical results for limbal transplants [50] because there are currently no suitable LESC markers. Quantitative analysis of mosaic corneal epithelial patterns in mouse X-inactivation mosaics and chimeras also suggests that *Pax6^+/−^* mice have fewer active clones of LESCs than normal [51]. However, the stripe pattern is disrupted, implying that cell movement is abnormal so *Pax6^+/−^* corneal deterioration probably involves additional factors.

Over-expression of Pax6 in hemizygous *PAX77^Tg/−^* mice with 5–7 copies of human *PAX6* [8] also causes eye abnormalities on a wild-type (WT) background and provides a model for human *PAX6* gene duplication [7]. The abnormalities overlap with those produced by heterozygous *Pax6^+/−^* mice (low Pax6 levels) but there are significant differences and genetic background modulates the phenotype [8], [52]–[55]. Pax6 levels are increased less than gene copy numbers predict [52], [54], [55] but the *PAX77* transgene can rescue various *Pax6^+/−^* and *Pax6^−/−^* ocular phenotypes [8].

The present study had three aims. (1) To investigate the effects of altered Pax6 dose on the corneal stroma and endothelium in *Pax6^+/−^* and *PAX77^Tg/−^* mice using EM, to complement previous studies of effects on the corneal epithelium. (2) Given that our previous clonal analysis of X-inactivation mosaics identified abnormalities of corneal epithelial maintenance in *Pax6^+/−^* mice [51], to investigate whether Pax6 over-expression causes similar abnormalities in *PAX77^Tg/−^* mice. (3) To investigate whether this effect can be rescued by combining the *PAX77* transgene with a *Pax6^+/−^* genotype. We identified previously unreported corneal endothelial and stromal abnormalities in both genotypes by EM. Furthermore, our qualitative analysis of X-inactivation mosaics implied that cell movement was normal during corneal epithelial maintenance in *PAX77^Tg/−^* mice (unlike *Pax6^+/−^* heterozygotes) and our quantification suggested that in younger mice LESC clone numbers were reduced in both *Pax6^+/−^* and *PAX77^Tg/−^* genotypes. The *PAX77* transgene normalised both the qualitative and quantitative defects in *Pax6^+/−^* corneas.

## Results

We used scanning electron microscopy (SEM) and transmission electron microscopy (TEM) to compare the structure of corneas from WT, *Pax6^+/−^* and *PAX77^Tg/−^* mice. We also analysed mosaic patterns in the corneal epithelia of WT, *Pax6^+/−^* and *PAX77^Tg/−^* X-inactivation mosaics to compare effects of Pax6 doses on corneal epithelial cell movement (from mosaic patterns) and LESC clone numbers (from quantitative analysis of corrected stripe numbers).

### *PAX77^Tg/−^* corneal epithelial cells have abnormally large microvilli and less pronounced cell junctions

**Figure 1:**
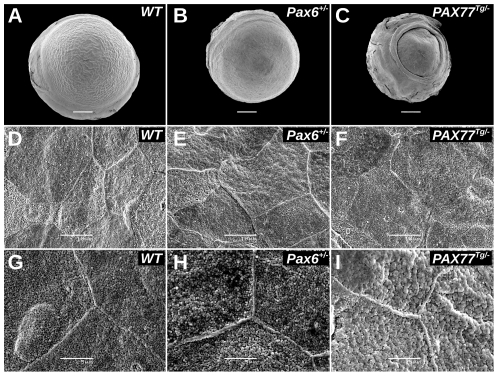
Electron microscopy of whole eyes and corneal epithelium. A–C. SEM micrographs of (A) WT, (B) *Pax6^+/−^* and (C) *PAX77^Tg/−^* eyes showing differences in size and gross morphology. Scale bars = 500 µm. (Diameters of WT, *Pax6^+/−^* and *PAX77^Tg/−^* eyes used for EM were approximately 2.9, 2.5 and 2.2 mm respectively and corneal diameters (dome base diameters) were approximately 2.5, 2.3 and 1.6 mm respectively.) D–F. SEM micrographs of (D) WT, (E) *Pax6^+/−^* and (F) *PAX77^Tg/−^* surfaces of corneal epithelial cells showing polygonal cell shapes and cell junctions. The surface of the cells appears more irregular in the *PAX77^Tg/−^* corneas than WT. Scale bars = 10 µm. G–I. Higher power SEM micrographs of (G) WT, (H) *Pax6^+/−^* and (I) *PAX77^Tg/−^* surfaces showing microvilli on cell surfaces. Microvilli are larger in the *PAX77^Tg/−^* corneas than WT. Scale bars = 5 µm.

On the CBA/Ca background *Pax6^+/−^* and *PAX77^Tg/−^* eyes were smaller than WT (Fig. 1A–C) and *PAX77^Tg/−^* mice had microcorneas and a pronounced ring around the corneoscleral junction (Fig. 1C). SEM of the superficial corneal epithelial cells showed that, the WT corneal epithelium consisted of large polygonal cells with tightly opposed cell junctions (Fig. 1D) and numerous microvilli (Fig. 1G). Despite reported increased sloughing of *Pax6^+/−^* corneal epithelial cells [38], the epithelia of the *Pax6^+/−^* specimens analysed by SEM appeared similar to WT (Fig. 1E,H). However, the *PAX77^Tg/−^* epithelial cells had a more irregular surface (Fig. 1F,I), indistinct cell junctions (Fig. 1F) and larger microvilli (Fig. 1I). Other corneal epithelial abnormalities have been well described, so further EM work focused on the corneal stroma and endothelium. Previously unreported abnormalities are discussed below and summarised in Table 1.

**Table 1:** Main new abnormalities in *Pax6^+/−^* and *PAX77^Tg/−^* corneas identified by electron microscopy.

Feature	Wild-type	*Pax6^+/−^*	*PAX77^Tg/−^*
Size of microvilli on surface of epithelial cells	normal	normal	large
Intracellular vacuoles in stromal keratocytes	none	yes (large)	yes (small)
Presence of nerve cells in stroma	present	more frequent*	more frequent*
Intracellular vacuoles in corneal endothelium	no	yes	yes
Corneal endothelial cell size and shape	regular/hexagonal	large/irregular	indistinct boundaries

*Nerve cell numbers appeared more frequent in *Pax6^+/−^* and *PAX77^Tg/−^* corneal stromas but they were not quantified.

### *Pax6^+/−^* and *PAX77^Tg/−^* corneal endothelial cells are severely abnormal

**Figure 2:**
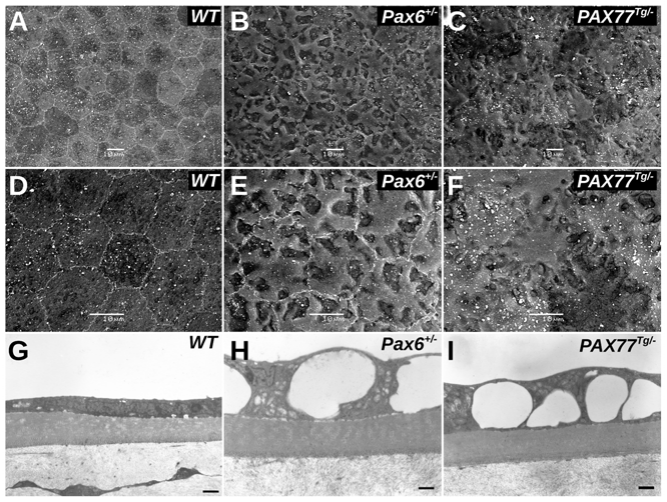
Electron microscopy of corneal endothelium. A–C. SEM micrographs of (A) WT, (B) *Pax6^+/−^* and (C) *PAX77^Tg/−^* corneal endothelial cells. WT endothelial cells have a regular hexagonal shape, *Pax6^+/−^* cells have an irregular vacuolated appearance and are larger than normal and *PAX77^Tg/−^* endothelial cells have an irregular vacuolated appearance, are irregular in shape and the cell borders are difficult to resolve. Scale bars = 10 µm. D–F. Higher power SEM micrographs of (D) WT, (E) *Pax6^+/−^* and (F) *PAX77^Tg/−^* corneal endothelial cells showing that although both *Pax6^+/−^* and *PAX77^Tg/−^* corneal endothelial cells are vacuolated and irregular in shape the cell borders are more distinct in *Pax6^+/−^* cells. Scale bars = 10 µm. G–I. TEM micrographs of (G) WT, (H) *Pax6^+/−^* and (I) *PAX77^Tg/−^* corneal endothelial cells (shown above Descemet's membrane and stroma) at the periphery of the cornea. *Pax6^+/−^* and *PAX77^Tg/−^* endothelial cells contain large vacuoles. Scale bars = 1 µm.

SEM examination revealed serious abnormalities in both *Pax6^+/−^* and *PAX77^Tg/−^* corneal endothelia. The WT corneal endothelial cells were hexagonal (mean diameter, 18.7±2.35 µm; Fig. 2A,D) whereas *Pax6^+/−^* cells were larger (23.75±3 µm; Fig. 2B,E) and slightly irregularly shaped. The *PAX77^Tg/−^* endothelium was highly irregular with indistinct cell borders that were only visible at higher magnification (Fig. 2C,F). The WT endothelial cells had either no vacuoles or very small vacuoles (Fig. 2D,G), whereas the *Pax6^+/−^* and *PAX77^Tg/−^* endothelial surfaces appeared highly irregular by SEM (Fig. 2E,F) and TEM revealed large intracellular vacuoles across the entire endothelium in each case (Fig. 2H,I). In *PAX77^Tg/−^* corneal endothelia the vacuoles seemed smaller towards the central cornea (data not shown). These results imply that Pax6 over- and under- expression both produced significant endothelial defects but over-expression produced a more severe phenotype.

### *Pax6^+/−^* and *PAX77^Tg/−^* corneal stromas are abnormal

**Figure 3:**
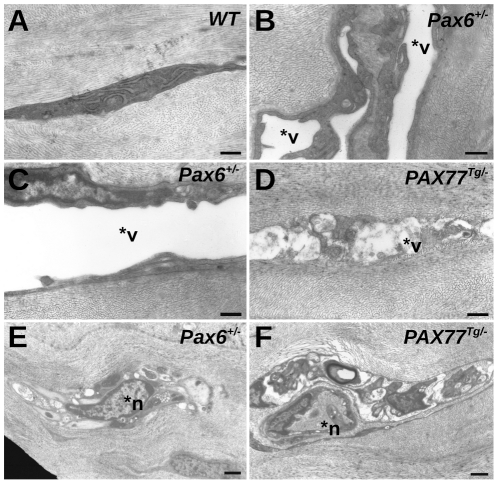
Electron microscopy of corneal stroma. TEM micrographs of (A) WT corneal stroma showing normal keratocyte morphology with no vacuoles; (B,C) *Pax6^+/−^* corneal stroma showing keratocytes with very large vacuoles; (D) *PAX77^Tg/−^* corneal stroma showing keratocyte with small vacuoles; (E) *Pax6^+/−^* corneal stroma showing nerve cell; (F) *PAX77^Tg/−^* corneal stroma showing nerve cell. Abbreviations: *v, vacuole; *n, nerve cell. Scale bars = 500 nm in A–D and 1 µm in E and F.

Marked abnormalities occurred in the corneal stromas of both the *Pax6^+/−^* and *PAX77^Tg/−^* mice. TEM showed that keratocytes in the WT stroma were normal with numerous cell organelles (Fig. 3A) but *Pax6^+/−^* keratocytes had large intracellular vacuoles (Fig. 3B,C) and *PAX77^Tg/−^* keratocytes had smaller vacuoles (Fig. 3D). Subjectively both *Pax6^+/−^* and *PAX77^Tg/−^* stromas also appeared to be more highly innervated with nerve cells (Fig. 3E,F) than in the WT stroma but this was not quantified.

### Over-expression of Pax6 in *PAX77^Tg/−^* mice does not disrupt corneal epithelial maintenance

**Figure 4:**
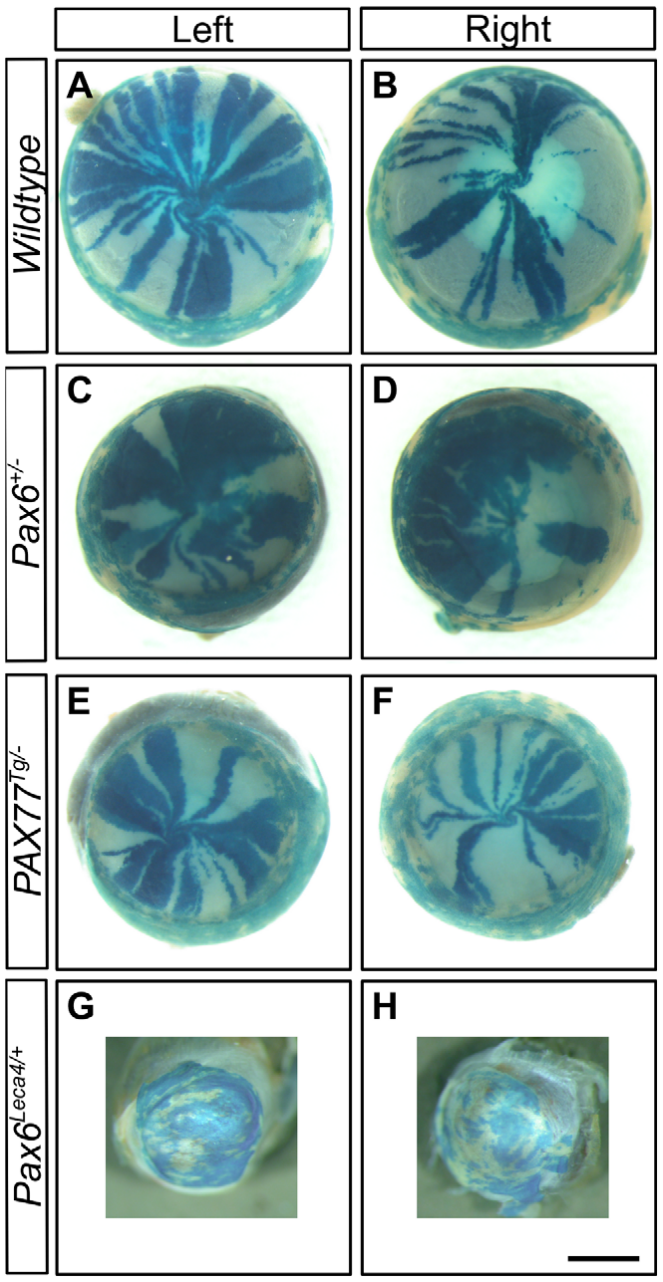
Mosaic patterns in the corneal epithelium. Representative images of β-gal staining in the corneal epithelia of X-inactivation mosaic eyes showing variation in mosaic patterns among the four different genotypes: (A,B) WT, *XLacZ^Tg/−^*; (C,D) *Pax6^+/−^*, *XLacZ^Tg/−^*; (E,F) *PAX77^Tg/−^*, *XLacZ^Tg/−^* and (G,H) *Pax6^Leca4/+^*, *XLacZ^Tg/−^*. Scale bar = 1 mm.

To compare the effects of different Pax6 doses on corneal epithelial maintenance by analysis of striped patterns in X-inactivation mosaics, we crossed *PAX77^Tg/−^* and *Pax6^+/−^* females to *XLacZ^Tg/Y^* males carrying the X-linked *LacZ* transgene and analysed patterns in the corneal epithelia of *XLacZ^Tg/−^* X-inactivation mosaic females, as described elsewhere [19]–[21]. WT, *XLacZ^Tg/−^* mosaics corneas showed radial striping patterns (Fig. 4A,B) whereas *Pax6^+/−^, XLacZ^Tg/−^* mosaics produced more irregular patterns (Fig. 4C,D) as demonstrated previously [19], [21], [51]. Strikingly, despite corneal defects (Tables 1 and 2), the *PAX77^Tg/−^*, *XLacZ^Tg/−^* mosaic eyes exhibited a qualitatively normal striped phenotype with radial stripes extending from the limbal region (Fig. 4E,F), consistent with normal centripetal movement from the presumptive LESCs. In contrast, the *Pax6^Leca4/+^, XLacZ^Tg/−^* mosaic corneal patterns appeared as an abnormal mosaic patchwork rather than radial stripes (Fig. 4G,H), so were not included in the quantitative analyses of stripe numbers described below.

**Table 2:** Comparison of maintenance of corneal epithelium in adult *Pax6^+/−^* and *PAX77^Tg/−^* mice with low and high doses of Pax6 respectively.

Feature	*Pax6^+/−^*	*PAX77^Tg/−^*
Number of corneal epithelial cell layers	Reduced [37], [38]	slightly reduced [54]
Fragility of corneal epithelium	fragile [38]	moderately fragile [56]
Cell proliferation in basal corneal epithelium	increased [38], [39]	increased [54]
Corneal opacity and vascularisation	yes [37], [43]	no [54], [55]
Abnormal corneal epithelial wound healing	yes [40], [42], [54]	yes [54]
Goblet cells in basal corneal epithelium	present [37]	absent [54], [55]
Mosaic pattern in corneal epithelia (15 & 30 weeks)	disrupted [20] *	normal*
Stripe number in mosaic corneal epithelia (15 weeks)	reduced [20] *	reduced*
Stripe number in mosaic corneal epithelia (∼30 weeks)	reduced (28 wks) [20] or normal (30 wks)*	normal*

*Reported in the present study.

**Figure 5:**
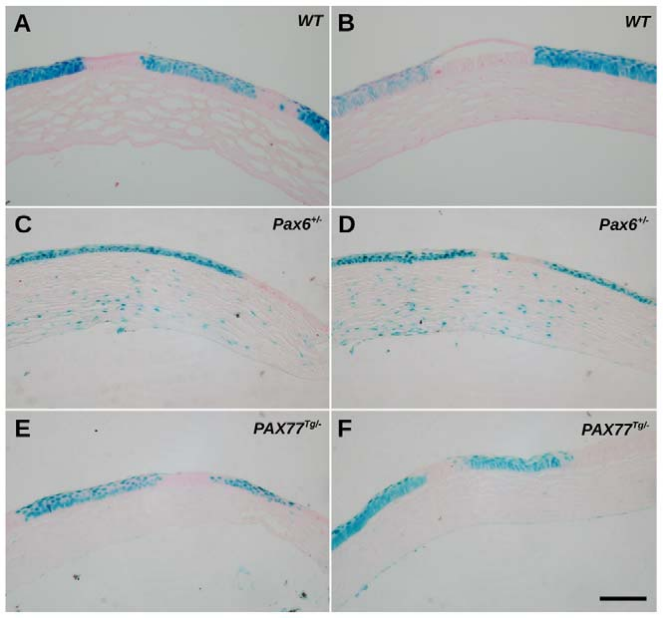
Sections of β-gal-stained mosaic corneal epithelia. Representative images of β-gal-stained corneal sections showing vertical alignment of β-gal-positive epithelial cells across the full thickness of the epithelium in eyes expressing various levels of Pax6. (A,B) WT, *XLacZ^Tg/−^*; (C,D) *Pax6^+/−^*, *XLacZ^Tg/−^* and (E,F) *PAX77^Tg/−^*, *XLacZ^Tg/−^*. The higher frequency of β-gal-positive cells in *XLacZ^Tg/−^*, *Pax6^+/−^* corneal stromas (C,D) probably reflects the greater permeability of the thin *Pax6^+/−^* corneal epithelium to X-gal stain during whole-mount staining. Scale bar = 100 µm.

In sections of stained *XLacZ^Tg/−^* corneas from WT, *Pax6^+/−^* and *PAX77^Tg/−^* animals, clones of β-gal positive cells were aligned vertically with little overlap of β-gal positive and β-gal negative cells (Fig. 5). We, therefore, treated the stripes as 2-dimensional patterns and reduced them to a 1-dimensional count for quantification (see Materials and Methods).

### Over-expression of Pax6 in *PAX77^Tg/−^* mosaic corneas results in fewer corneal stripes

Although stripe patterns in *PAX77^Tg/−^, XLacZ^Tg/−^* mosaics appeared normal, a quantitative analysis was undertaken to identify any subtle differences that might suggest that stem cell clones were affected. The observed number of radial stripes in the corneal epithelium was converted to a corrected stripe number to compare LESC clone numbers between different groups (see Materials and Methods). A preliminary experiment, using *PAX77^Tg/−^* mice on an outbred CD-1 genetic background showed that the corrected stripe number per eye was significantly lower in *PAX77^Tg/−^, XLacZ^Tg/−^* mosaics than WT, *XLacZ^Tg/−^* controls at 15 weeks (Table 3). This difference remained significant after correcting for the smaller circumferences of *PAX77^Tg/−^* corneas, suggesting that *PAX77^Tg/−^, XLacZ^Tg/−^* corneas were maintained by fewer active LESC clones than normal.

**Table 3:** Preliminary comparison of corrected stripe number in corneal epithelia of wild-type, *XLacZ^Tg/−^* and *PAX77^Tg/−^, XLacZ^Tg/−^* X-inactivation mosaics at 15 weeks.

	Wild-type	*PAX77^Tg/−^*	Significance (*t*-test)
Number of eyes	27	16	
Corrected stripe number per eye (mean ±SEM)	79.97±3.65	54.07±3.43	*P*<0.0001
Corrected stripe number per mm corneal circumference	7.65±0.35	5.93±0.45	*P* = 0.005

Although the corrected stripe number does not provide a direct estimate of LESC numbers, it can be used to compare LESC clones between different groups of mice. The corrected stripe number is an estimate of the number of corneal epithelial clones, each of which will be produced by an active coherent clone of LESCs in the limbus. A difference in corrected stripe number, therefore, predicts a difference in the number of active LESC clones and this could reflect a difference in active LESC numbers and/or a change in the LESC distribution (number of LESCs per clone).

**Figure 6:**
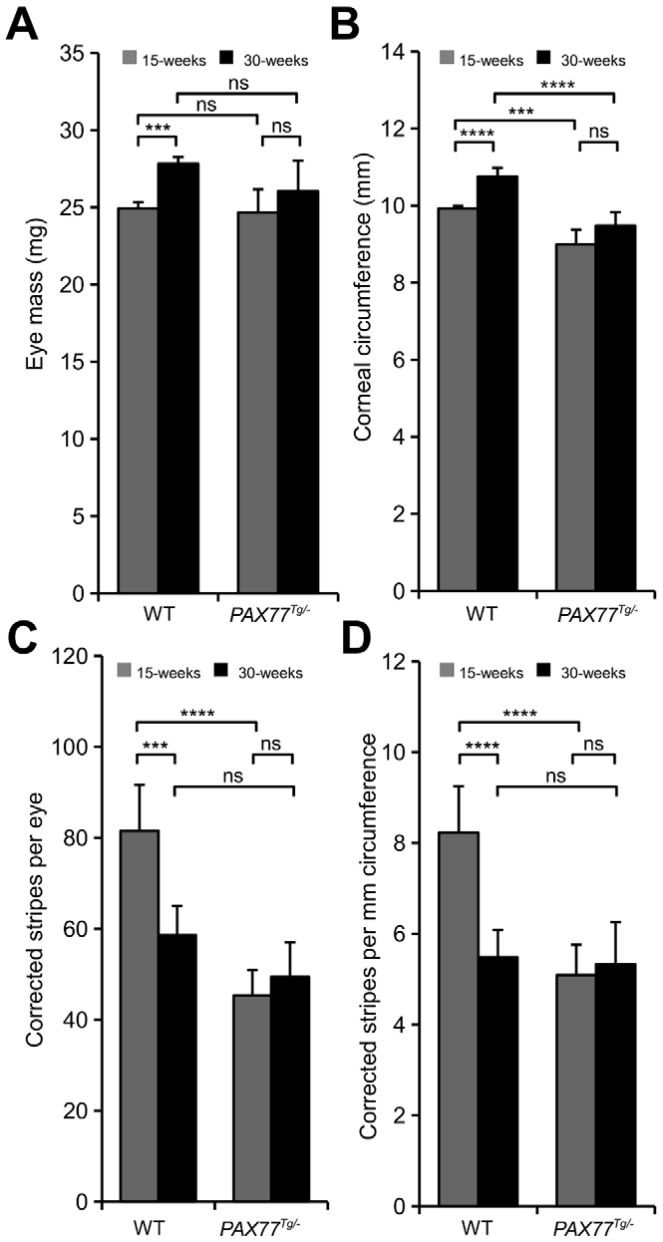
Quantitative comparisons of *PAX77^Tg/−^ XLacZ^Tg/−^* and *PAX77^−/−^ XLacZ^Tg/−^* eyes at 15 and 30-weeks. (A) WT Eye mass increased significantly between 15 and 30 weeks (2-way ANOVA *P*<0.0001, results of relevant post-hoc tests are shown in the figure). (B) Corneal circumference differed significantly between WT and *PAX77^Tg/−^* at 15 and 30 weeks but the increase in circumference between 15 and 30 weeks was only significant for WT (2-way ANOVA *P*<0.0001, results of relevant post-hoc tests are shown in the figure). (C) The mean corrected stripe number was significantly higher in the 15-week WT corneas than the 30-week WT or 15-week *PAX77^Tg/−^* groups, there was a significant decline in stripe number between 15 and 30 weeks in the WT but not the *PAX77^Tg/−^* group (2-way ANOVA *P*<0.0001, results of relevant post-hoc tests are shown in the figure). (D) The mean corrected stripe number was significantly higher in the 15-week WT corneas than the 30-week WT or 15-week *PAX77^Tg/−^* groups, there was a significant decline in stripe number between 15 and 30 weeks in the WT but not the *PAX77^Tg/−^* group (2-way ANOVA *P*<0.0001, results of relevant post-hoc tests are shown in the figure). For each comparison there were 14–36 eyes per group: 22 15-week WT, 36 30-week WT, 14 15-week *PAX77^Tg/−^* and 20 30-week *PAX77^Tg/−^*. Significant *P*-values for Tukey's HSD post-hoc tests are shown: ns = not significant; *P<0.05; **P<0.01; ***P<0.001; ****P<0.0001. For all post-hoc tests see Tables S1, S2, S3, S4.

The preliminary results were confirmed and extended in a second experiment, using *PAX77^Tg/−^* mice on an inbred CBA/Ca genetic background and analysing corneas at both 15 and 30 weeks. The mean corrected stripe number per eye (or per mm circumference) was significantly lower in *PAX77^Tg/−^, XLacZ^Tg/−^* mosaics than WT, *XLacZ^Tg/−^* controls at 15 weeks (Fig. 6). It declined between 15 and 30 weeks in WT, *XLacZ^Tg/−^* controls, as reported previously [19], [21], but no reduction occurred in the *PAX77^Tg/−^, XLacZ^Tg/−^* mosaics, so by 30 days the *PAX77^Tg/−^* corrected stripe number was not significantly different from controls (Fig. 6C,D). This suggests that *PAX77^Tg/−^, XLacZ^Tg/−^* corneas were maintained by fewer active LESC clones than normal at 15 weeks (as in the preliminary experiment, Table 3) but, unlike WT, this did not decline further between 15 and 30 weeks.

### The *PAX77* transgene normalises corneal circumference and corneal epithelial maintenance in *Pax6^+/−^* heterozygotes

**Figure 7:**
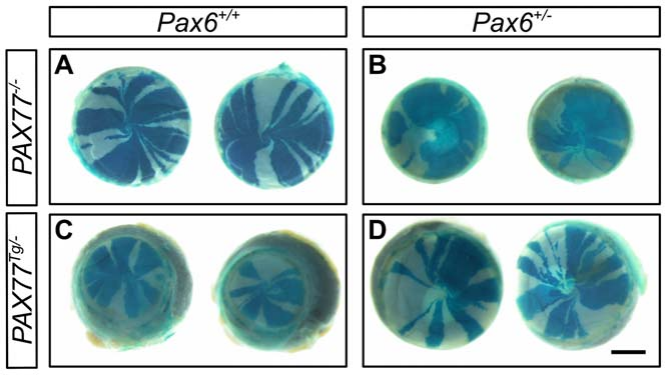
Representative images of β-gal staining in the corneal epithelia of X-inactivation mosaic eyes expressing different levels of Pax6. (A) WT (*PAX77^−/−^, Pax6^+/+^, XLacZ^Tg/−^*) eyes exhibit ordered radial stripes of clonally related epithelial cells. (B) *PAX77^−/−^, Pax6^+/−^, XLacZ^Tg/−^* eyes are smaller and striping patterns are disrupted, normal radial stripes are only rarely observed. (C) In eyes over-expressing PAX6 (*PAX77^Tg/−^*, *Pax6^+/+^*, *XLacZ^Tg/−^*) the corneal epithelial diameter is smaller in comparison to the overall eye size (microcornea) but normal radial stripe patterns are visible. (D) *PAX77^Tg/−^*, *Pax6^+/−^*, *XLacZ^Tg/−^* corneas appear normal both in size and striping phenotype. Scale bar = 1 mm.

**Figure 8:**
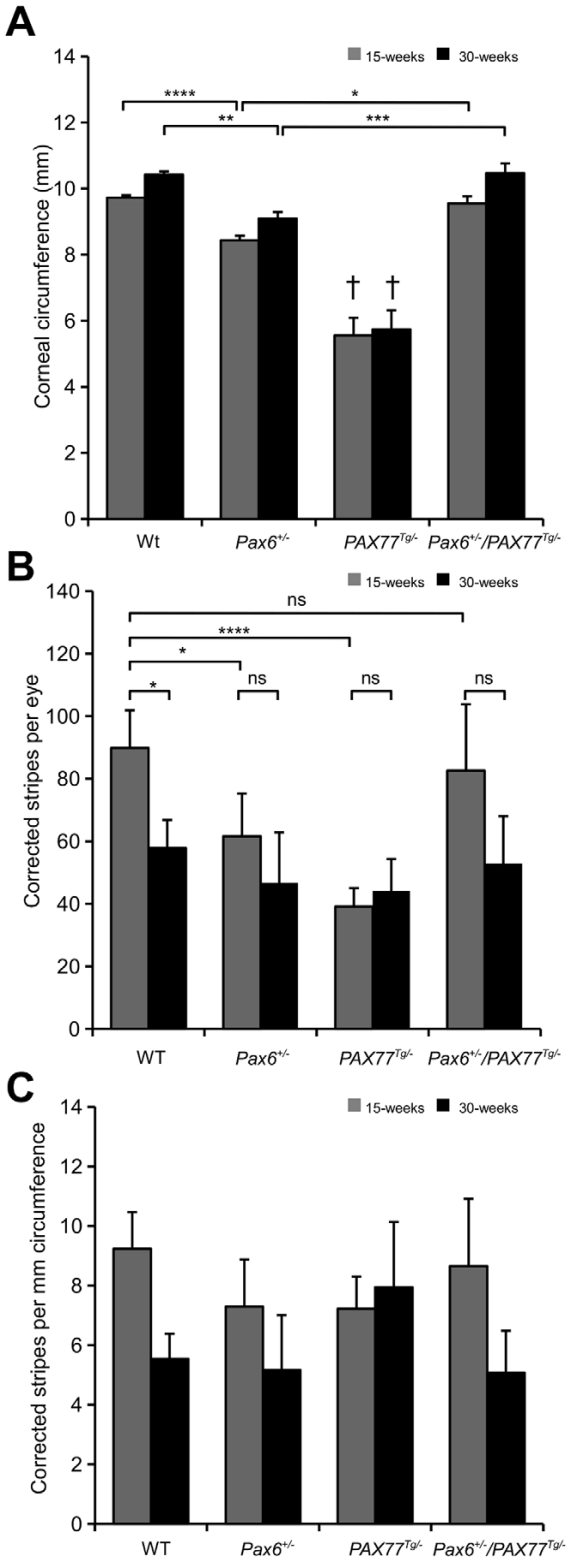
Quantitative comparison of stripe patterns in corneal epithelia of X-inactivation mosaics of four genotypes. Corneal circumferences and corrected stripe numbers were compared in WT (*PAX77^−/−^*, *Pax6^+/+^*, *XLacZ^Tg/−^*); *Pax6^+/−^* (*PAX77^−/−^*, *Pax6^+/−^*, *XLacZ^Tg/−^*); *PAX77^Tg/−^* (*PAX77^Tg/−^*, *Pax6^+/+^*, *XLacZ^Tg/−^*); and combined *PAX77^Tg/−^*, *Pax6^+/−^* (*PAX77^Tg/−^*, *Pax6^+/−^*, *XLacZ^Tg/−^*) mosaic eyes at both 15 and 30-weeks. (A) The corneal circumference was significantly smaller in both 15-week and 30-week old *PAX77^Tg/−^* (*Pax6^+/+^*, *PAX77^Tg/−^*, *XLacZ^Tg/−^*) mice than in the three other genotypes at both ages (2-way ANOVA *P*<0.0001, results of relevant post-hoc tests are shown in the figure). (B) The *Pax6^+/−^, PAX77^Tg/−^* corrected stripe number did not differ from WT at 15 weeks. The WT corrected stripe number declined significantly between 15 and 30 weeks. This was not the case for any other group. (2-way ANOVA *P*<0.0001, results of relevant post-hoc tests are shown in the figure). (C) On this genetic background correcting the mean corrected stripe number for circumference abrogated the significant results observed in B (2-way ANOVA *P*<0.01, but results of relevant post-hoc tests were all non-significant). For each comparison there were 7–25 eyes per group: 25 15-week WT, 12 30-week WT, 20 15-week *Pax6^+/−^*, 12 30-week *Pax6^+/−^*, 7 15-week *PAX77^Tg/−^*, 16 30-week *PAX77^Tg/−^*, 22 15-week *Pax6^+/+^, PAX77^Tg/−^* and 22 30-week *Pax6^+/+^, PAX77^Tg/−^* eyes. Significant *P*-values for Tukey's HSD post-hoc tests are shown: ns = not significant; *P<0.05; **P<0.01; ***P<0.001; ****P<0.0001. † *P*<0.0001 for differences with all other genotypes at both ages. For all post-hoc tests see Tables S5, S6, S7.

The additional Pax6 expression produced by the *PAX77^Tg/−^* transgene can rescue abnormal ocular phenotypes in *Pax6^+/−^* mice [8]. To determine whether the *PAX77^Tg/−^* transgene could also rescue the abnormal *Pax6^+/−^* striped pattern in mosaics (implying abnormal corneal epithelial maintenance; Fig. 4E,F), we undertook a third experiment which compared striping patterns in the four *XLacZ^Tg/−^* mosaic genotypes produced by crosses of *Pax6^+/−^* females and *PAX77^Tg/−^*, *XLacZ^Tg/Y^* males. The stripe patterns for the three genotypes already examined in earlier experiments (Fig. 4A–F) were reproduced at both 15 and 30 weeks in the third experiment (Fig. 7A–C) although on the genetic background produced by this three-way cross, mice over-expressing Pax6 (*PAX77^Tg/−^*, *Pax6^+/+^, XLacZ^Tg/−^*) had smaller corneas (Fig. 7C; compare Figs. 6B and 8A). In *PAX77^Tg/−^, Pax6^+/−^, XLacZ^Tg/−^* mosaics, where the *PAX77* transgene is expressed in a *Pax6^+/−^* genetic background, the corneal circumference was larger than in *Pax6^+/−^, XLacZ^Tg/−^* and *PAX77^Tg/−^, XLacZ^Tg/−^* mosaics and not significantly different from WT (Fig. 8A). Similarly, in *PAX77^Tg/−^, Pax6^+/−^, XLacZ^Tg/−^* mosaic eyes the striping pattern appeared qualitatively normal (Fig. 7D), unlike the disrupted *Pax6^+/−^* pattern (Fig. 7B). This indicates that normal centrifugal corneal epithelial movement had been restored.

### The *PAX77* transgene restores corneal epithelial stripe numbers in *Pax6^+/−^, PAX77^Tg/−^* mice

Although the mosaic corneal stripe patterns appeared normal in *PAX77^Tg/−^, Pax6^+/−^, XLacZ^Tg/−^* mosaics (Fig. 7), the second experiment shows that this may mask quantitative differences (Fig. 6C,D). Thus, to investigate whether the striped pattern was normalised quantitatively as well as qualitatively by the presence of the *PAX77* transgene in *PAX77^Tg/−^, Pax6^+/−^, XLacZ^Tg/−^* mosaics, we analysed the corrected stripe number per cornea for the four genotypes at 15 and 30 weeks of age (Fig. 8). At 15 weeks, corrected stripe numbers in *PAX77^Tg/−^, Pax6^+/−^, XLacZ^Tg/−^* mosaics were not significantly different from WT, *XLacZ^Tg/−^* mosaics, but were significantly higher than in either *PAX77^−/−^, Pax6^+/−^*, *XLacZ^Tg/−^* or *PAX77^Tg/−^, Pax6^+/+^*, *XLacZ^Tg^* ^/−^ mosaics (Fig. 8B). The corrected stripe number later declined in both *PAX77^Tg/−^, Pax6^+/−^, XLacZ^Tg/−^* and WT, *XLacZ^Tg/−^* mosaics and by 30 weeks there were no significant differences among the four genotypes. When results were expressed as corrected stripes per mm of corneal circumference (Fig. 8C), the *PAX77^Tg/−^, Pax6^+/−^, XLacZ^Tg/−^* and WT, *XLacZ^Tg/−^* mosaics again had more stripes than the other genotypes at 15 weeks but these differences failed to reach significance. Nevertheless, corrected stripe numbers in *PAX77^Tg/−^, Pax6^+/−^, XLacZ^Tg/−^* mosaics were quantitatively similar to those in WT, *XLacZ^Tg/−^* mosaics, suggesting that the *PAX77* transgene had rescued the low 15-week *Pax6^+/−^* stripe number (estimated LESC clone number) as well as the qualitative mosaic pattern.

## Discussion

This study identified new corneal abnormalities, particularly in the stroma and endothelium, in both *Pax6^+/−^* and *PAX77^Tg/−^* mice, which respectively under- and over-express Pax6. Analysis of mosaic corneal patterns showed cell movement during corneal epithelial maintenance was affected in *Pax6^+/−^* but not *PAX77^Tg/−^* mice and implied that LESC clones were affected in both *Pax6^+/−^* and *PAX77^Tg/−^* mice at 15 weeks.

### Morphological abnormalities of *Pax6^+/−^* and *PAX77^Tg/−^* corneas

The abnormally large microvilli and indistinct cell junctions of *PAX77^Tg/−^* corneal epithelial cells are consistent with fragility tests suggesting cell adhesion may be affected [56]. Significant new morphological abnormalities were identified in the corneal stroma and endothelium of both *Pax6^+/−^* and *PAX77^Tg/−^* mice including intracellular vacuoles. There is some evidence that Pax6 is expressed weakly and transiently in the mouse fetal corneal stroma [47], [57] and chimera experiments imply that Pax6 functions cell-autonomously in developing stromal keratocytes or their progenitors [57]. However, it is not known whether the keratocyte and endothelial abnormalities described here are primary defects of altered Pax6 dose in these lineages or an indirect effect mediated via another tissue. The corneal endothelium controls corneal hydration and nutrition via fluid transport. Excessive hydration may cause corneal stromal haze or corneal oedema, as in some human corneal stromal dystrophies. Similar corneal endothelial and stromal keratocyte vacuolation also occurs in human macular corneal dystrophy [58], [59]. If the corneal endothelium and/or stroma of *PAX6* ^+/−^ human aniridia patients are affected like the *Pax6* ^+/−^ mice described here, this might have important clinical implications and could underlie some of the abnormal phenotypes associated with aniridia-related keratopathy.

### Effects of Pax6 on corneal epithelial cell movement and maintenance

The normal radial striped corneal epithelial pattern of WT X-inactivation mosaics was disrupted in *Pax6^+/−^* mosaics, implying that corneal epithelial cell movement is abnormal [51] but it is unclear whether this is caused by some intrinsic abnormality or a response to chronic wounding of a thin and fragile cornea. Although maintenance of the *PAX77^Tg/−^* corneal epithelium is not entirely normal (Table 2), *PAX77^Tg/−^* mosaics had completely normal radial striped patterns, implying that centripetal cell movement is unaffected by the higher Pax6 dose. In contrast, the pattern in corneas of *Pax6^Leca4/+^* mosaics resembled the randomly orientated pattern of patches seen in young WT corneas before stripes emerge. This suggests that movement of cells from the limbus is severely reduced and that the *Pax6^Leca4/+^*corneal epithelium may be maintained by proliferation from within the epithelium, perhaps because of an extreme LESC deficiency. This possibility has previously been suggested to explain a similar phenotype in *Dstn^corn1/corn1^* homozygotes [60].

### Evidence for effects of Pax6 dose on limbal stem cell clones

Our quantitative analysis of striped patterns in *Pax6^+/−^* and *PAX77^Tg/−^* X-inactivation mosaics showed that, at 15 weeks, the corrected stripe numbers were lower than in WT mosaics, even after correcting for differences in corneal circumference, implying that at this age there were fewer clones of active LESCs maintaining the corneal epithelium. However, no such difference was seen at 30 weeks because, by this age, the stripe number had declined in WT mosaics but the stripe numbers did not decline between 15 and 30 weeks in *Pax6^+/−^* and *PAX77^Tg/−^* mosaics.

The decline in corrected stripe number in WT *XLacZ* mosaics implies that there is an age-related decline in active LESC clones. This may reflect a decline in LESC numbers or activity but it could also be explained by a drift in LESC clone distributions. If LESCs can divide either symmetrically to produce one LESC and one TAC or asymmetrically to produce either two LESCs or two TACs then the pattern of active stem cell clones may follow a pattern of neutral drift as suggested for some other stem cell systems, including spermatogonial stem cells [61] and intestinal crypts [62], [63]. Over time, stem cells may be lost and replaced by their neighbours. On this basis, clones of stem cells will expand and contract stochastically and some clones will be lost (e.g. if a β-gal negative LESC clone, that is flanked by two β-gal positive clones, is lost the β-gal negative stripe will be lost and the two flanking β-gal positive stripes will merge into one larger one).

The lower corrected stripe number in *Pax6^+/−^* and *PAX77^Tg/−^ XLacZ* mosaics compared to WT mosaics at 15 weeks can be explained in several ways. It is possible that there are initially fewer LESC clones in *Pax6^+/−^* and *PAX77^Tg/−^* eyes, either because fewer LESCs are specified, or activated or because the LESCs are grouped into fewer larger clones. Alternatively, LESC clone numbers may initially be similar in all groups (before 15 weeks) but the decline may begin earlier or be more rapid in *Pax6^+/−^* and *PAX77^Tg/−^* mice, so by 15 weeks the LESC clone number is similar to that in a 30 week WT mouse. It is not clear why the *Pax6^+/−^* and *PAX77^Tg/−^* LESC clone numbers do not continue to decline after 15 weeks, but it has been suggested that this might be related to a minimum required for corneal epithelial maintenance [21]. Regardless of the explanation it is clear that, at 15 weeks, LESC clones numbers and/or distributions are different in *Pax6^+/−^* and *PAX77^Tg/−^* mice. However, as this difference is no longer detectable at 30 weeks the difference is short-lived and may have little biological significance.

The presence of goblet cells in the human corneal epithelium is often cited as evidence of LESC deficiency [49], [64]. However, the quantitative analysis of *Pax6^+/−^* and *PAX77^Tg/−^* mosaics indicates LESC clones are similarly affected in both genotype at 15 weeks (Fig. 8B,C) but only *Pax6^+/−^* mice have goblet cells in the corneal epithelium (Table 2). This difference highlights the need for reliable LESC markers.

### The *PAX77* transgene compensates for defects of corneal maintenance in *Pax6^+/−^* X-inactivation mosaics

On a *Pax6^+/−^* background, the *PAX77* transgene rescued the abnormal stripe patterns that normally occur in *Pax6^+/−^* heterozygotes and are attributed to low Pax6 levels. Quantitative analysis showed that the *PAX77* transgene also normalised the putative deficiency in active stem cell clones (reduced stripe number) that occurs in *Pax6^+/−^* heterozygotes at 15 weeks. These results imply that restoring the Pax6 dose to a more normal level corrects abnormalities of corneal cell maintenance as well as the developmental ocular defects demonstrated previously [8].

It is widely believed that stem cell deficiency causes most corneal abnormalities in ARK. However, our quantitative analyses of mosaic patterns suggest that *Pax6^+/−^* and *PAX77^Tg/−^* mice have only relatively modest reductions in LESC clone numbers. In contrast, both *Pax6^+/−^* and *PAX77^Tg/−^* mice have severe corneal endothelial and stromal defects. This should prompt further investigations of the pathophysiology underlying ARK.

## Materials and Methods

Consumables were purchased from Sigma (Poole, UK) and procedures carried out at room temperature, unless stated otherwise.

### Ethics statement

All animal work was approved by a University of Edinburgh internal ethics committee and was performed in accordance with institutional guidelines under license by the UK Home Office (project license number PPL 60/3635).

### Animals and genetic crosses

Mice were maintained in animal facilities of the College of Medicine and Veterinary Medicine, University of Edinburgh. Heterozygous *Pax6^+/Sey-Neu^* mice (abbreviated to *Pax6^+/−^*) and wild-type (WT, *Pax6^+/+^*) littermates were produced from *Pax6^+/+^* female × *Pax6^+/−^* male crosses on a CBA/Ca genetic background and genotyped by PCR as described previously [3]. Heterozygous *Pax6^Leca4/+^* mice, on a mixed genetic background, were provided by Prof. Ian Jackson and Dr Sally Cross (MRC, Human Genetics Unit, Edinburgh) and maintained as a closed, random-bred colony by crossing *Pax6^Leca4/+^* and WT mice within the colony. Outbred CD-1 mice carrying the *PAX77* transgene which expresses 5–7 copies of the human *PAX6* gene [8] were provided by Professor Veronica van Heyningen and Dr Dirk A. Kleinjan (MRC Human Genetics Unit, Edinburgh) and the transgene was transferred to the inbred CBA/Ca strain by genetic crosses as reported previously [55]. In the present study we designated mice hemizygous for the *PAX77* transgene as *PAX77^Tg/−^* (because the use of ‘*Tg*’ to designate presence of the transgene is less ambiguous than ‘+’ used in our previous ‘*PAX77^+/−^*’ notation [55]) and we designate non-transgenic littermates as *PAX77^−/−^*. The founder colony is designated CD1-*PAX77^Tg^* and was maintained by CD-1 × CD1-*PAX77^Tg/−^* crosses. The derived congenic stock is designated CBA-*PAX77^Tg^* and was maintained by CBA/Ca × CBA-*PAX77^Tg/−^* crosses. Hemizygous *PAX77^Tg/−^* mice and WT, *PAX77^−/−^* littermates were genotyped by PCR as described previously [55]. No homozygous *PAX77^Tg/Tg^* transgenic mice were used in this study.

H253 strain mice [65], ubiquitously expressing the *Tg(Hmgcr-lacZ)H253Sest*, X-linked *nLacZ* transgene (abbreviated to *XLacZ*), were obtained from the MRC Mammalian Genetics Unit, Harwell, UK, as strain FTH, and maintained on a genetic background that was predominantly a mixture of C57BL/6 and CBA/Ca inbred strains. Males and females hemizygous for this X-linked transgene are designated respectively *XLacZ^Tg/Y^* and *XLacZ^Tg/−^*; female homozygotes are designated *XLacZ^Tg/Tg^*. X-inactivation mosaics hemizygous for the *Pax6^Sey-Neu^* null mutation and the *Pax6^Leca4^* missense mutation, plus WT littermate controls, were produced from *Pax6^+/−^* female × *XLacZ^Tg/Y^* male and *Pax6^Leca4/+^* female × *XLacZ^Tg/Y^* male crosses respectively.

In a preliminary experiment, *PAX77^Tg/−^, XLacZ^Tg/−^* and WT control *PAX77^−/−^, XLacZ^Tg/−^* X-inactivation mosaic females were produced using the original CD1-*PAX77^Tg^* stock in *XLacZ^Tg/Tg^* female × CD1-*PAX77^Tg/−^* male and CD1-*PAX77^Tg/−^* female × *XLacZ^Tg/Y^* male crosses. Once the *PAX77^Tg/−^* transgene had been bred onto the CBA/Ca genetic background, female *PAX77^Tg/−^, XLacZ^Tg/−^* and *PAX77^Tg/−^, XLacZ^Tg/−^* littermates were produced from crosses between CBA-*PAX77^Tg/−^* females and hemizygous *XLacZ^Tg/Y^* males for a second experiment. In a third experiment, crosses between *Pax6^+/−^* females and hemizygous CBA-*PAX77^Tg/−^, XLacZ^Tg/Y^* males were used to produce 4 types of *XLacZ^Tg/−^*, X-inactivation mosaic females: (1) *PAX77^Tg/−^, Pax6^+/−^, XLacZ^Tg/−^*; (2) *PAX77^Tg/−^, Pax6^+/+^*, *XLacZ^Tg/−^*; (3) *PAX77^−/−^, Pax6^+/−^*, *XLacZ^Tg/−^* and (4) *PAX77^−/−^, Pax6^+/+^*, *XLacZ^Tg/−^*.

### Electron microscopy

Eyes from adult (8–22 weeks old) *Pax6^+/Sey-Neu^* (*Pax6^+/−^*) and WT (*Pax6^+/+^*) littermates plus *PAX77^Tg/−^* and WT (*PAX77^−/−^*) littermates, all on a CBA/Ca genetic background, were enucleated and fixed in 2.5% (w/v) glutaraldehyde in 0.1 M sodium cacodylate buffer prior to processing for scanning electron microscopy as described elsewhere [59]. Whole eyes were washed three times in cacodylate buffer for 15 minutes. Samples were post-fixed in 2% (w/v) osmium tetroxide for 3 hours and washed again in cacodylate buffer before being passed through a graded ethanol series.

For scanning electron microscopy (SEM), samples were transferred to hexamethyldisilazane (HMDS) for 40 minutes and air-dried. The samples were mounted on aluminium stubs and sputter coated with gold using an Edwards S150A sputter coater then examined on a JEOL JSM 5600 scanning electron microscope.

For transmission electron microscopy (TEM), samples were transferred to propylene oxide twice for 20 minutes each time. They were placed in a solution containing 50% propylene oxide and 50% Araldite resin (Agar Scientific, UK) overnight, after which they were transferred to 100% resin and infiltrated overnight under agitation. The samples were embedded in moulds containing fresh resin and polymerised at 60°C for 24–36 hours. Ultra-thin sections (50–70 nanometres thick) were cut on a Reichert Ultracut E microtome, collected on naked copper grids and counterstained for 1 hour each with 1% vanadyl sulphate and phosphotungstic acid and then 15 minutes with Reynolds' lead citrate prior to examination on a JEOL JEM 1010 transmission electron microscope.

### Clonal analysis of X-inactivation striping patterns

X-gal staining of *XLacZ^Tg/−^* eyes and the acquisition of images have been described previously [19], [21]. Striping patterns were analysed automatically as described in Mort [66]. Photographs of eyes were taken so that the entire cornea was visible and were then cropped to the edge of the corneal epithelium and analysed using ImageJ, a freeware software package designed by Wayne Rasband for the National Institute of Health (NIH), USA (http://rsb.info.nih.gov/ij/). The observed number of radial stripes in the corneal epithelium was corrected for the probability that stripes would contain multiple adjacent β-gal-positive corneal epithelial clones. This involved dividing the observed mean width by the function 1/(1-*p*), where *p* is the proportion of β-gal-positive cells around the circumference as described previously [19]–[21]. The corrected stripe number provides an estimate of the total number of active corneal epithelial coherent clones (both β-gal positive and β-gal negative) per circumference. This is useful for comparing numbers of active clones of stem cells between different groups but because the number of stem cells per coherent clone may vary it is not a direct estimate of the number of active stem cells. For the preliminary experiment mosaic corneal patterns were analysed manually at 15 weeks using Adobe Photoshop software as described previously [19]. For later mosaic analyses performed at both 15 and 30 weeks, the ImageJ plugin ‘Clonal Tools’ [66] was used in batch mode to analyse all the images automatically. Where correction for the actual circumference was required this was calculated by dividing the number of corrected stripes by the circumference of each eye measured using ImageJ.

### Histology

Whole eyes dissected at 15 and 30 weeks after birth were fixed and stained for β-gal reporter activity using X-gal as described previously [19], [21]. X-gal stained eyes were embedded in paraffin wax and 7 µm sections were cut on a microtome, mounted on standard microscope slides and counterstained with eosin and neutral red as described previously [21].

### Statistics

2-way ANOVAs and Tukey's HSD post-hoc tests were calculated using R statistical software (http://www.r-project.org/). Student's t-tests were calculated using Microsoft Excel.

## Supporting Information

Table S1 **Multiple comparisons of** ***PAX77^Tg/−^*** **and** ***PAX77^−/−^*** **eyes mass.** (See Fig. 6A.)
(PDF)

Table S2 **Multiple comparisons of** ***PAX77^Tg/−^*** **and** ***PAX77^−/−^*** **corneal circumference.** (See Fig. 6B.)
(PDF)

Table S3 **Multiple comparisons of** ***PAX77^Tg/−^*** **and** ***PAX77^−/−^*** **corrected stripe number.** (See Fig. 6C.)
(PDF)

Table S4 **Multiple comparisons of** ***PAX77^Tg/−^*** **and** ***PAX77^−/−^*** **corrected stripe number per mm circumference.** (See Fig. 6C.)
(PDF)

Table S5 **Multiple comparisons of** ***WT, Pax6^+/−^*** **,** ***PAX77^Tg/−^*** **and** ***Pax6^+/−^ PAX77^Tg/−^*** **corneal circumference.** (See Fig. 8A.)
(PDF)

Table S6 **Multiple comparisons of** ***WT, Pax6^+/−^*** **,** ***PAX77^Tg/−^*** **and** ***Pax6^+/−^ PAX77^Tg/−^*** **corrected stripe number.** (See Fig. 8B.)
(PDF)

Table S7 **Multiple comparisons of** ***WT, Pax6^+/−^*** **,** ***PAX77^Tg/−^*** **and** ***Pax6^+/−^ PAX77^Tg/−^*** **corrected stripe number per mm circumference.** (See Fig. 8C.)
(PDF)
