# Supplementary material for: Effects of Aberrant Pax6 Gene Dosage on Mouse Corneal Pathophysiology and Corneal Epithelial Homeostasis
Source: PLoS One. 2011 Dec 29;6(12):e28895. doi: 10.1371/journal.pone.0028895 (PMC3248408; doi:10.1371/journal.pone.0028895)
Supplement: Table S1 — Multiple comparisons of PAX77Tg/− and PAX77−/− eyes mass. (See Fig. 6A.) (PDF) [file pone.0028895.s001.pdf]

Table S1: Multiple comparisons of *PAX77*$^{Tg/-}$ and *PAX77*$^{-/-}$ eyes mass (See Fig. 6A)

Group A	Group B	Tukey HSD P value
PAX77$^{Tg/-}$ 30 weeks	*PAX77*$^{Tg/-}$ 15 weeks	0.4088
WT 15 weeks	*PAX77*$^{Tg/-}$ 15 weeks	0.9887
WT 30 weeks	*PAX77*$^{Tg/-}$ 15 weeks	**0.0010**
WT 15 weeks	*PAX77*$^{Tg/-}$ 30 weeks	0.5014
WT 30 weeks	*PAX77*$^{Tg/-}$ 30 weeks	0.0678
WT 30 weeks	*WT* 15 weeks	**0.0004**
