# Supplementary material for: Effects of Aberrant Pax6 Gene Dosage on Mouse Corneal Pathophysiology and Corneal Epithelial Homeostasis
Source: PLoS One. 2011 Dec 29;6(12):e28895. doi: 10.1371/journal.pone.0028895 (PMC3248408; doi:10.1371/journal.pone.0028895)
Supplement: Table S2 — Multiple comparisons of PAX77Tg/− and PAX77−/− corneal circumference. (See Fig. 6B.) (PDF) [file pone.0028895.s002.pdf]

Table S2: Multiple comparisons of $PAX77^{Tg/-}$ and $PAX77^{-/-}$ corneal circumference (See Fig. 6B)

Group A	Group B	Tukey HSD P value
$PAX77^{Tg/-}$ 30 weeks	$PAX77^{Tg/-}$ 15 weeks	0.1357
WT 15 weeks	$PAX77^{Tg/-}$ 15 weeks	**0.0003**
WT 30 weeks	$PAX77^{Tg/-}$ 15 weeks	**<0.0001**
WT 15 weeks	$PAX77^{Tg/-}$ 30 weeks	0.1208
WT 30 weeks	$PAX77^{Tg/-}$ 30 weeks	**<0.0001**
WT 30 weeks	*WT* 15 weeks	**<0.0001**
