# Supplementary material for: Effects of Aberrant Pax6 Gene Dosage on Mouse Corneal Pathophysiology and Corneal Epithelial Homeostasis
Source: PLoS One. 2011 Dec 29;6(12):e28895. doi: 10.1371/journal.pone.0028895 (PMC3248408; doi:10.1371/journal.pone.0028895)
Supplement: Table S5 — Multiple comparisons of WT, Pax6+/− , PAX77Tg/− and Pax6+/− PAX77Tg/− corneal circumference. (See Fig. 8A.) (PDF) [file pone.0028895.s005.pdf]

Table S5: Multiple comparisons of *WT, Pax6$^{+/-}$*, *PAX77$^{Tg/-}$* and *Pax6$^{+/-}$PAX77$^{Tg/-}$* corneal circumference (See Fig. 8A)

Group A	Group B	Tukey HSD P value
Pax6$^{+/-}$ PAX77$^{-/-}$ 30 weeks	*Pax6$^{+/-}$ PAX77$^{-/-}$* 15 weeks	0.3495
Pax6$^{+/-}$ PAX77$^{Tg/-}$ 15 weeks	*Pax6$^{+/-}$ PAX77$^{-/-}$* 15 weeks	**0.0416**
Pax6$^{+/-}$ PAX77$^{Tg/-}$ 30 weeks	*Pax6$^{+/-}$ PAX77$^{-/-}$* 15 weeks	**<0.0001**
Pax6$^{+/+}$ PAX77$^{Tg/-}$ 15 weeks	*Pax6$^{+/-}$ PAX77$^{-/-}$* 15 weeks	**<0.0001**
Pax6$^{+/+}$ PAX77$^{Tg/-}$ 30 weeks	*Pax6$^{+/-}$ PAX77$^{-/-}$* 15 weeks	**<0.0001**
WT 15 weeks	*Pax6$^{+/-}$ PAX77$^{-/-}$* 15 weeks	**<0.0001**
WT 30 weeks	*Pax6$^{+/-}$ PAX77$^{-/-}$* 15 weeks	**<0.0001**
Pax6$^{+/-}$ PAX77$^{Tg/-}$ 15 weeks	*Pax6$^{+/-}$ PAX77$^{-/-}$* 30 weeks	0.9292
Pax6$^{+/-}$ PAX77$^{Tg/-}$ 30 weeks	*Pax6$^{+/-}$ PAX77$^{-/-}$* 30 weeks	**0.0004**
Pax6$^{+/+}$ PAX77$^{Tg/-}$ 15 weeks	*Pax6$^{+/-}$ PAX77$^{-/-}$* 30 weeks	**<0.0001**
Pax6$^{+/+}$ PAX77$^{Tg/-}$ 30 weeks	*Pax6$^{+/-}$ PAX77$^{-/-}$* 30 weeks	**<0.0001**
WT 15 weeks	*Pax6$^{+/-}$ PAX77$^{-/-}$* 30 weeks	0.3300
WT 30 weeks	*Pax6$^{+/-}$ PAX77$^{-/-}$* 30 weeks	**0.0023**
Pax6$^{+/-}$ PAX77$^{Tg/-}$ 30 weeks	*Pax6$^{+/-}$ PAX77$^{Tg/-}$* 15 weeks	0.2025
Pax6$^{+/+}$ PAX77$^{Tg/-}$ 15 weeks	*Pax6$^{+/-}$ PAX77$^{Tg/-}$* 15 weeks	**<0.0001**
Pax6$^{+/+}$ PAX77$^{Tg/-}$ 30 weeks	*Pax6$^{+/-}$ PAX77$^{Tg/-}$* 15 weeks	**<0.0001**
WT 15 weeks	*Pax6$^{+/-}$ PAX77$^{Tg/-}$* 15 weeks	0.9996
WT 30 weeks	*Pax6$^{+/-}$ PAX77$^{Tg/-}$* 15 weeks	0.3230
Pax6$^{+/+}$ PAX77$^{Tg/-}$ 15 weeks	*Pax6$^{+/-}$ PAX77$^{Tg/-}$* 30 weeks	**<0.0001**
Pax6$^{+/+}$ PAX77$^{Tg/-}$ 30 weeks	*Pax6$^{+/-}$ PAX77$^{Tg/-}$* 30 weeks	**<0.0001**
WT 15 weeks	*Pax6$^{+/-}$ PAX77$^{Tg/-}$* 30 weeks	0.0876
WT 30 weeks	*Pax6$^{+/-}$ PAX77$^{Tg/-}$* 30 weeks	1.0000
Pax6$^{+/+}$ PAX77$^{Tg/-}$ 30 weeks	*Pax6$^{+/+}$ PAX77$^{Tg/-}$* 15 weeks	0.9949
WT 15 weeks	*Pax6$^{+/+}$ PAX77$^{Tg/-}$* 15 weeks	**<0.0001**
WT 30 weeks	*Pax6$^{+/+}$ PAX77$^{Tg/-}$* 15 weeks	**<0.0001**
WT 15 weeks	*Pax6$^{+/+}$ PAX77$^{Tg/-}$* 30 weeks	**<0.0001**
WT 30 weeks	*Pax6$^{+/+}$ PAX77$^{Tg/-}$* 30 weeks	**<0.0001**
WT 30 weeks	*WT* 15 weeks	0.2290
