# Supplementary material for: Effects of Aberrant Pax6 Gene Dosage on Mouse Corneal Pathophysiology and Corneal Epithelial Homeostasis
Source: PLoS One. 2011 Dec 29;6(12):e28895. doi: 10.1371/journal.pone.0028895 (PMC3248408; doi:10.1371/journal.pone.0028895)
Supplement: Table S6 — Multiple comparisons of WT, Pax6+/− , PAX77Tg/− and Pax6+/− PAX77Tg/− corrected stripe number. (See Fig. 8B.) (PDF) [file pone.0028895.s006.pdf]

Table S6: Multiple comparisons of *WT, Pax6*$^{+/-}$, *PAX77*$^{Tg/-}$ and *Pax6*$^{+/-}$*PAX77*$^{Tg/-}$ corrected stripe number (See Fig. 8B)

Group A	Group B	Tukey HSD P value
Pax6$^{+/-}$ *PAX77*$^{-/-}$ 30 weeks	*Pax6*$^{+/-}$ *PAX77*$^{-/-}$ 15 weeks	0.7717
Pax6$^{+/-}$ *PAX77*$^{Tg/-}$ 15 weeks	*Pax6*$^{+/-}$ *PAX77*$^{-/-}$ 15 weeks	0.6086
Pax6$^{+/-}$ *PAX77*$^{Tg/-}$ 30 weeks	*Pax6*$^{+/-}$ *PAX77*$^{-/-}$ 15 weeks	0.9746
Pax6$^{+/+}$ *PAX77*$^{Tg/-}$ 15 weeks	*Pax6*$^{+/-}$ *PAX77*$^{-/-}$ 15 weeks	0.1101
Pax6$^{+/+}$ *PAX77*$^{Tg/-}$ 30 weeks	*Pax6*$^{+/-}$ *PAX77*$^{-/-}$ 15 weeks	0.3824
WT 15 weeks	*Pax6*$^{+/-}$ *PAX77*$^{-/-}$ 15 weeks	**0.0108**
WT 30 weeks	*Pax6*$^{+/-}$ *PAX77*$^{-/-}$ 15 weeks	0.9999
Pax6$^{+/-}$ *PAX77*$^{Tg/-}$ 15 weeks	*Pax6*$^{+/-}$ *PAX77*$^{-/-}$ 30 weeks	0.0856
Pax6$^{+/-}$ *PAX77*$^{Tg/-}$ 30 weeks	*Pax6*$^{+/-}$ *PAX77*$^{-/-}$ 30 weeks	0.9985
Pax6$^{+/+}$ *PAX77*$^{Tg/-}$ 15 weeks	*Pax6*$^{+/-}$ *PAX77*$^{-/-}$ 30 weeks	0.9928
Pax6$^{+/+}$ *PAX77*$^{Tg/-}$ 30 weeks	*Pax6*$^{+/-}$ *PAX77*$^{-/-}$ 30 weeks	1.0000
WT 15 weeks	*Pax6*$^{+/-}$ *PAX77*$^{-/-}$ 30 weeks	**0.0002**
WT 30 weeks	*Pax6*$^{+/-}$ *PAX77*$^{-/-}$ 30 weeks	0.9666
Pax6$^{+/-}$ *PAX77*$^{Tg/-}$ 30 weeks	*Pax6*$^{+/-}$ *PAX77*$^{Tg/-}$ 15 weeks	0.2058
Pax6$^{+/+}$ *PAX77*$^{Tg/-}$ 15 weeks	*Pax6*$^{+/-}$ *PAX77*$^{Tg/-}$ 15 weeks	**0.0050**
Pax6$^{+/+}$ *PAX77*$^{Tg/-}$ 30 weeks	*Pax6*$^{+/-}$ *PAX77*$^{Tg/-}$ 15 weeks	**0.0210**
WT 15 weeks	*Pax6*$^{+/-}$ *PAX77*$^{Tg/-}$ 15 weeks	0.9980
WT 30 weeks	*Pax6*$^{+/-}$ *PAX77*$^{Tg/-}$ 15 weeks	0.4978
Pax6$^{+/+}$ *PAX77*$^{Tg/-}$ 15 weeks	*Pax6*$^{+/-}$ *PAX77*$^{Tg/-}$ 30 weeks	0.7513
Pax6$^{+/+}$ *PAX77*$^{Tg/-}$ 30 weeks	*Pax6*$^{+/-}$ *PAX77*$^{Tg/-}$ 30 weeks	0.9710
WT 15 weeks	*Pax6*$^{+/-}$ *PAX77*$^{Tg/-}$ 30 weeks	**0.0006**
WT 30 weeks	*Pax6*$^{+/-}$ *PAX77*$^{Tg/-}$ 30 weeks	0.9997
Pax6$^{+/+}$ *PAX77*$^{Tg/-}$ 30 weeks	*Pax6*$^{+/+}$ *PAX77*$^{Tg/-}$ 15 weeks	0.9984
WT 15 weeks	*Pax6*$^{+/+}$ *PAX77*$^{Tg/-}$ 15 weeks	**<0.0001**
WT 30 weeks	*Pax6*$^{+/+}$ *PAX77*$^{Tg/-}$ 30 weeks	0.4932
WT 15 weeks	*Pax6*$^{+/+}$ *PAX77*$^{Tg/-}$ 30 weeks	**<0.0001**
WT 30 weeks	*Pax6*$^{+/+}$ *PAX77*$^{Tg/-}$ 15 weeks	0.8275
WT 30 weeks	*WT* 15 weeks	**0.0155**
