# Supplementary material for: Effects of Aberrant Pax6 Gene Dosage on Mouse Corneal Pathophysiology and Corneal Epithelial Homeostasis
Source: PLoS One. 2011 Dec 29;6(12):e28895. doi: 10.1371/journal.pone.0028895 (PMC3248408; doi:10.1371/journal.pone.0028895)
Supplement: Table S7 — Multiple comparisons of WT, Pax6+/− , PAX77Tg/− and Pax6+/− PAX77Tg/− corrected stripe number per mm circumference. (See Fig. 8C.) (PDF) [file pone.0028895.s007.pdf]

Table S7: Multiple comparisons of *WT, Pax6*$^{+/-}$, *PAX77*$^{Tg/-}$ and *Pax6*$^{+/-}$*PAX77*$^{Tg/-}$ corrected stripe number per mm circumference (See Fig. 8C)

Group A	Group B	Tukey HSD P value
Pax6$^{+/-}$ *PAX77*$^{-/-}$ 30 weeks	*Pax6*$^{+/-}$ *PAX77*$^{-/-}$ 15 weeks	0.6947
Pax6$^{+/-}$ *PAX77*$^{Tg/-}$ 15 weeks	*Pax6*$^{+/-}$ *PAX77*$^{-/-}$ 15 weeks	0.9856
Pax6$^{+/-}$ *PAX77*$^{Tg/-}$ 30 weeks	*Pax6*$^{+/-}$ *PAX77*$^{-/-}$ 15 weeks	0.5425
Pax6$^{+/+}$ *PAX77*$^{Tg/-}$ 15 weeks	*Pax6*$^{+/-}$ *PAX77*$^{-/-}$ 15 weeks	1.0000
Pax6$^{+/+}$ *PAX77*$^{Tg/-}$ 30 weeks	*Pax6*$^{+/-}$ *PAX77*$^{-/-}$ 15 weeks	0.9987
WT 15 weeks	*Pax6*$^{+/-}$ *PAX77*$^{-/-}$ 15 weeks	0.5703
WT 30 weeks	*Pax6*$^{+/-}$ *PAX77*$^{-/-}$ 15 weeks	0.8614
Pax6$^{+/-}$ *PAX77*$^{Tg/-}$ 15 weeks	*Pax6*$^{+/-}$ *PAX77*$^{-/-}$ 30 weeks	0.4030
Pax6$^{+/-}$ *PAX77*$^{Tg/-}$ 30 weeks	*Pax6+/- PAX77*$^{-/-}$ 30 weeks	1.0000
Pax6$^{+/+}$ *PAX77*$^{Tg/-}$ 15 weeks	*Pax6+/- PAX77*$^{-/-}$ 30 weeks	0.7117
Pax6$^{+/+}$ *PAX77*$^{Tg/-}$ 30 weeks	*Pax6*$^{+/-}$ *PAX77*$^{-/-}$ 30 weeks	0.3346
WT 15 weeks	*Pax6*$^{+/-}$ *PAX77*$^{-/-}$ 30 weeks	**0.0224**
WT 30 weeks	*Pax6*$^{+/-}$ *PAX77*$^{-/-}$ 30 weeks	1.0000
Pax6$^{+/-}$ *PAX77*$^{Tg/-}$ 30 weeks	*Pax6*$^{+/-}$ *PAX77*$^{Tg/-}$ 15 weeks	0.3069
Pax6$^{+/+}$ *PAX77*$^{Tg/-}$ 15 weeks	*Pax6*$^{+/-}$ *PAX77*$^{Tg/-}$ 15 weeks	0.9794
Pax6$^{+/+}$ *PAX77*$^{Tg/-}$ 30 weeks	*Pax6*$^{+/-}$ *PAX77*$^{Tg/-}$ 15 weeks	0.9997
WT 15 weeks	*Pax6*$^{+/-}$ *PAX77*$^{Tg/-}$ 15 weeks	0.9999
WT 30 weeks	*Pax6*$^{+/-}$ *PAX77*$^{Tg/-}$ 15 weeks	0.5558
Pax6$^{+/+}$ *PAX77*$^{Tg/-}$ 15 weeks	*Pax6*$^{+/-}$ *PAX77*$^{Tg/-}$ 30 weeks	0.5573
Pax6$^{+/+}$ *PAX77*$^{Tg/-}$ 30 weeks	*Pax6*$^{+/-}$ *PAX77*$^{Tg/-}$ 30 weeks	0.1935
WT 15 weeks	*Pax6*$^{+/-}$ *PAX77*$^{Tg/-}$ 30 weeks	**0.0060**
WT 30 weeks	*Pax6*$^{+/-}$ *PAX77*$^{Tg/-}$ 30 weeks	1.0000
Pax6$^{+/+}$ *PAX77*$^{Tg/-}$ 30 weeks	*Pax6*$^{+/+}$ *PAX77*$^{Tg/-}$ 15 weeks	0.9971
WT 15 weeks	*Pax6*$^{+/+}$ *PAX77*$^{Tg/-}$ 15 weeks	0.4902
WT 30 weeks	*Pax6*$^{+/+}$ *PAX77*$^{Tg/-}$ 30 weeks	0.8753
WT 15 weeks	*Pax6*$^{+/+}$ *PAX77*$^{Tg/-}$ 30 weeks	0.9039
WT 30 weeks	*Pax6*$^{+/+}$ *PAX77*$^{Tg/-}$ 15 weeks	0.5297
WT 30 weeks	*WT* 15 weeks	0.0554
